# Protein kinase inhibitors for acute leukemia

**DOI:** 10.1186/s40364-018-0123-1

**Published:** 2018-02-13

**Authors:** Yuan Ling, Qing Xie, Zikang Zhang, Hua Zhang

**Affiliations:** Guangdong Provincial Key Laboratory of Medical Molecular Diagnostics, Institute of Laboratory Medicine, Guangdong Medical University, Dongguan, 523808 China

**Keywords:** Protein kinase inhibitor, Acute lymphocyte leukemia, Acute myeloid leukemia

## Abstract

Conventional treatments for acute leukemia include chemotherapy, radiation therapy, and intensive combined treatments (including bone marrow transplant or stem cell transplants). Novel treatment approaches are in active development. Recently, protein kinase inhibitors are on clinical trials and offer hope as new drugs for acute leukemia treatment. This review will provide a brief summary of the protein kinase inhibitors in clinical applications for acute leukemia treatment.

## Background

Acute leukemia is subdivided into acute myelocytic leukemia (AML) and acute lymphoblastic leukemia (ALL) [1, 2]. AML accounts for about 90% of all acute leukemias in adults, and the cure rates are 35–40% in patients under 60 years old and 5–15% in those over 60 years old respectively [3]. The elderly always have poor prognosis with a median survival of 5–10 months. ALL is the most common subtype found in childhood with a peak incidence in 2–5 years. Although more than 80% of children with ALL receive positive effect after treatments, there are only 20%–40% of adults ALL [4]. Philadelphia-positive acute lymphoblastic leukemia (Ph + ALL) has poor prognosis which is the most frequent genetic subtype of adult ALL and, in the elderly, Ph + ALL accounts for approximately 30% of cases [5, 6]. To date, chemotherapy is still the main treatment strategy for leukemia. Although hematopoietic stem cell transplantation (HSCT) is also sometimes used as front-line therapy for patients with high-risk leukemia, usually, it is considered when induction chemotherapy fails or leukemia relapses [7, 8]. Cancer cells typically evade the immune surveilence and have genetic heterogeneity with mutant targets [9]. Currently, emerging molecular targeted therapy is being used in clinic, such as inhibitors of FMS-like tyrosine kinase 3 (FLT3) and mammalian target of rapamycin (mTOR) in acute leukemia [10]. Besides, new inhibitors specific to novel targets like IDH1/2, PP2A, DOCK2, PAK1 have been developed [11].

Thus, targeted inhibitors have been developed as replacements for conventional chemotherapy and provide a less toxic and more effective way than the conventional chemotherapy. Here, we will provide a comprehensive overview of the main protein kinase inhibitors (PKIs) used or being developed in acute leukemia.

### Protein kinase inhibitors in acute leukemia

Protein kinases are conventionally divided into five classes: protein tyrosine kinase, protein serine/threonine kinase, tryptophan protein kinase, histidine protein kinase and protein aspartyl/glutamoyl kinase. It has been proved that the abnormal activity of protein kinases is associated with many diseases like, inflammation immune system disease, and cancer including leukemia [12]. The main protein kinases particularly involve the phosphatidyl-inositol 3-kinase/v-akt murine thymoma viral oncogene homolog 1 (PI3K/AKT), mitogenactivated protein kinase/extracellular signal regulated kinase (MAPK/ERK), janus kinase signal transducer and activator of transcription (JAK-STAT) and signal transducer and activator of transcription 5 (STAT5) in AML observed to be aberrantly activated in a variety of malignancies, including pre-B-ALL, T cell ALL, and AML [13, 14]. Other targets have been reported, such as FLT3, Bruton’s tyrosine kinase (BTK), mTOR, AKT, poly (ADP-ribose) polymerase (PARP), histone deacetylase (HDAC), etc. [15]. Thus, protein kinases have become new focus and PKIs have been developed as new anti-tumor drugs to disrupt the abnormal signal transduction in the therapy of acute leukemia.

As we all know the ABL-inhibitor imatinib became the first Food and Drug Administration (FDA)-approved small molecule protein kinase blocker. However, due to the emergence of many new mutation sites of protein kinase, the drug resistance to imatinib is more and more serious. Other pharmacological inhibitors including dasatinib and nilotinib, which are significantly more potent than imatinib and may overcome resistance have been developed. Imatinib and dasatinib, are registered for the treatment of Ph + ALL in adults [16]. On the other hand, imatinib and sunitinib reduce AML cell by blocking the activity of c-KIT pharmacologically [17, 18]. Many molecular changes are being studied the prognitic impact in acute leukemia. However, only FMS-like tyrosine kinase 3 internal tandem duplications (FLT3-ITDs), Nucleophosmin (NPM1), CCAAT/enhancer-binding protein-α (C/EBP-α) and c-KIT have been currently incorporated in validated international risk stratification schema [19]. FLT3-ITDs is associated with worse prognosis in AML and several FLT3 inhibitors have undergone clinical trials [20]. Here, we summarized some PKIs are being used or under clinical evaluation at phase I, II and III clinical trials in acute leukemia (Table 1).

**Table 1:** The therapeutic protein kinase inhibiors in acute leukemia

Targets	Inhibitors	Comments	Main side effects	Phase
FLT3	Quizartinib	An anti-FLT3 TKI, was investigated in ALL or AML	Nausea, anemia, vomiting, etc	I-II-III [70]
	Midostaurin	It is an oral multi-targeted kinase inhibitor to inihibit leukemia cells including ALL and FLT3-positive AML	Diarhhoea, nausea, headache, etc.	I-II-III [71]
	Sunitinib	Sunitinib inhibits leukemia cells survival and angiogenesis	Cardiotoxic, dyspnea, etc.	II [72]
	Lestaurtinib	Lestaurtinib might inhibit the activity of FLT3 kinase and it is more appropriate during intensive chemotherapy	Gastrointestinal reaction, etc.	I-II-III [73]
	Tandutinib	It inhibits the FLT3 ITD-positive rather than the ITD-negative patients with AML.	Bone pain, nausea, etc.	I-II-III
	Gilteritinib	Gilteritinib is a kind of favourable safety agent and is being on trial at 120 mg/day	Diarrhea, etc.	I-II -III
	Crenolanib	Crenolanib is a potential selective inhibitor of FLT3-ITDs and PDGFRα/β	Vomiting, headache, etc	II
	Bortezomib	It is associated with apoptotic and autophagic cell death of AML	Gastro-intestinal, asthenia, etc	I-II-III [74]
BTK	Ibrutinib	Although ibrutinib has its own unique toxicity, it usually causes fewer infections	Pneumonia, sinusitis, headache upper respiratory tract infection, etc.	II
	Acalabrutinib	It has received accelerated approval for the treatment of cell lymphoma	Headache, diarrhea, weight gain skin rash, severe diarrhea, etc.	I-II
JAK-STAT	Ruxolitinib	The JAK1/JAK2 inhibitor, is more effective against JAK-STAT pathway	Nausea, anemia, vomiting, ect.	III
	Pacritinib (SB1518)	It is potent inhibitor FLT3-ITDs, JAK2, JAK2V617F in phase III development	Anemia, ect.	I-II-III [75]
	Everolimus	Everolimus is combined with MK-2206, particularly important in the setting of resistance to therapeutic drugs	Cardiac failure, respiratory failure septic shock, etc.	I-II [76]
	Temsirolimus	Temsirolimus combines with etoposide, cyclophosphamide and dexamethasone for relapsed pediatric ALL in adults	Nausea, etc.	I-II [77]
mTOR	Sirolimus	Sirolimus is an mTOR inhibitor, but it has a similar suppressive effect on the immune system	Abdominal pain, nausea, etc.	II [78]
	AZD8055	AZD8055 inhibits the phosphorylation of mTORC1 with p70S6K and 4E–BP1 and downstream proteins	Anorexia, etc.	I
	Ciclopirox	Ciclopirox enhances the effect of the preclinical antileukemia while irritation	Itching, blistering, swelling, etc.	I
MEK	Pimasertib	It is a novel, selective, orally bioavailable MEK1/2	Bleeding risk, etc.	II
	GSK690693	It inhibits apoptosis in sensitive ALL cell lines	Not clearly	I
AKT	MK-2206	An orally inhibitor of the PI3K/Akt pathway which can inhibit tumor cell proliferation	Fatigue, vomiting, anorexia, etc	I-II
	T315I	The mutation of T315I occurs to patients even when second- and third-genneration on trails	Nausea, swelling, rash, etc.	II-III
	Gefitinib	A third-line agent and also is an EGFR inhibitor	Diarrhoea, vomiting, anorexia, etc.	II [79]
	Ponatinib	It is a multi-targeted tyrosine-kinase inhibitor often with hypertension	Hypertension, rash, abdominal pain, fatigue, etc.	I-II
Bcr-Abl	Dasatinib	It also inhibits the Src kinase family	Anemia, diarrhea, swelling, rash, etc	II
	ABL001	ABL001 is taken orally and the high does of it can be given safely to patients	Not clearly	I
	BEZ235	BEZ-235 is a PI3K inhibitor. It also inhibits mTOR. It is being investigated as a possible leukemia treatment.	Anemia, vomiting, etc	I-II
PI3K	Idelalisib	Idelalisib is effective in leukemia patients who have p53 mutation	Fever, fatigue, nausea, cough abdominal, pain, rash, chills, etc.	on trial
	PKI-587	Gedatolisib is an agent targeting the PI3K/mTOR pathway	Nausea, etc.	I
PLK1	Volasertib	It has been reported the volasertib inhibits PLK1 in both cancer and normal cells	Anaema, throm bocytopenia, nausea febrile neutropenia, etc.	I-II

## Tyrosine protein kinase inhibitors

### FLT3 inhibitors

#### Quizartinib (AC220)

Quizartinib is a second-generation FLT3 inhibitor and selectively inhibits class III receptor tyrosine kinases including FLT3, stem cell factor receptor (SCFR), colony-stimulating factor 1 receptor (CSF1R) and platelet derived growth factor receptors (PDGFRs), thus usually results in a better complete remissions in relapsed/refractory (r/r) AML [21]. Other studies also showed that quizartinib can lead to favorable prognosis for the treatment of AML [22, 23].

#### Midostaurin

Midostaurin, a multi-target protein kinase inhibitor with anti-FLT3 activity which also combined with conventional induction and consolidation therapies, has been found to significantly prolong survival of FLT3-mutated AML patients in the phase III clinical trial [15]. After successful Phase II clinical trials, midostaurin was approved by the FDA for the treatment of adult AML patients of FLT3 positive usually combining with chemotherapy or for companion diagnose to detect the FLT3 mutation in patients with AML [24]. Notably, the combination of midostaurin and chemotherapy for AML patients had 23% improvement in overall survival (OS) [25].

#### Sunitinib

Sunitinib (SU11248), the first FLT3 inhibitor studied in the clinic, plays a role in inhibiting both tumor angiogenesis and tumor cell proliferation, and usually is used with cytarabine against AML cell lines with FL3-ITD mutation. However, sunitinib is a multi-targeted receptor tyrosine kinase (RTK) inhibitor and was proved to target platelet-derived growth factor (PDGF-Rs), vascular endothelial growth factor receptors (VEGFRs), and CD117 (c-KIT) receptor tyrosine kinases except for FLT3 [26]. Sunitinib has been recommended as a second-line drug for patients resistant to imatinib due to c-KIT mutations or those who can not tolerate sunitinib [27].

#### Lestaurtinib

Lestaurtinib (rINN, codenamed CEP-701) is an inhibitor of the kinases FLT3, tropomyosin receptor kinase A/B/C (TrkA/B/C), Janus kinase 2 (JAK2) [28]. It is quite nonselective like inhibiting other tyrosine kinases like Janus kinase 2 (JAK2), and tropomycin receptor kinase (TRK) [29]. Previous studies have shown that lestaurtinib has the potential to promote apoptosis in FLT3-ITD leukemic cells [30]. Currently, lestaurtinib is being investigated to be combined with chemotherapy in infants and young children [31].

#### Tandutinib (MLN518)

Tandutinib, can supress the autophosphorylation of FLT3, c-KIT and PDGF (platelet-derived growth factor) receptor tyrosine kinases, thereby inhibiting cellular proliferation and inducing apoptosis in AML [32]. Tandutinib can improve the CR rate to 90% in primarily diagonosed AML patients when used with daunorubicin and cytarabine [33].

#### Gilteritinib (ASP2215)

Gilteritinib, a pyrazinecarboxamide derivative, is on trial at phase I-II-III and a promising novel inhibitor of FLT3 inhibitors for its potential activity against all classes of FLT3-activating mutations [34]. In addition, a trial of gilteritinib as maintenance therapy for the first remission patients receiving HSCT are to be investigated [35].

#### Crenolanib (PLX-3397)

Crenolanib, the third generation agent, is an orally available drug that selectively and potently inhibits signaling of class III receptor tyrosine kinases (RTK) FLT3 and platelet-derived growth factor receptor α/β (PDGFR α/β). Unlike most RTK inhibitors, crenolanib can be applied to the induction chemotherapy in AML [36].

#### Bortezomib (PS-341)

Bortezomib, a reversible 26S proteasome inhibitor, can interfere with the transcription and induce the early degradation of FLT3 internal tandem duplications (FLT3-ITD) through autophagy against ALL [37]. In addition, bortezomib can trigger the inhibition of MAPK/ERK, PI3K/AKT and STAT5 pathways in the AML [38, 39]. Evidence showed that bortezomib could potentiate the cytotoxic effects of combination chemotherapy in patients with leukemia.

### BTK inhibitors

#### Ibrutinib (Imbruvica)

Ibrutinib inhibits pre-BCR+ B-cell by targeting Bruton’s tyrosine kinase (BTK) and B lymphocyte kinase (BLK), while selectively targets FLT3-ITD in mutant FLT3-positive in AML [40]. Clinical trials studies have demonstrated its tolerability in malignant B cells and has progressed into phase III trials. The combination treatment of ibrutinib with vincristine or dexamethasone demonstrated valid activity during the therapy of ALL [41].

#### Acalabrutinib (ACP-196)

Acalabrutinib, a second generation BTK inhibitor, has been shown to be better than ibrutinib for improved targeting specificity for BTK [42]. Large clinical samples and longer follow-up are still needed to ascertain these potential advantages.

### JAK-STAT inhibitors

#### Ruxolitinib

The JAK-STAT cell signaling pathway mainly regulates gene transcription. And the combination of ruxolitinib with nilotinib usually inhibits the proliferation of leukemia cells especially in Ph + ALL [43].

#### Pacritinib (SB1518)

Pacritinib (SB1518) was found to inhibit JAK2/FLT3 with a great potential for its less side effects in advanced myeloid malignancies, myelofibrosis and myeloproliferative neoplasms during phase I/II study [44].

### Other inhibitors

#### Gefitinib (ZD1839)

Gefitinib is the third generation agent, and the first selective inhibitor of epidermal growth factor receptor (EGFR) tyrosine kinase domain. Although gefitinib can induce differentiation in AML cells, a phase II trial has shown that conventional use of gefitinib as a single agent for AML is not yet clear. Therefore, additional clinical trails are currently recruiting [45].

#### Ponatinib

Ponatinib (previously AP24534), the third-generation TKI, is an oral TKI drug developed for the treatment of T315I-positive Ph + ALL, however, it’s appilcation may be limited for some ALL patients particularly with imatinib-resistance and multiple mutations [46].

## Protein serine/threonine kinase inhibitors

### mTOR inhibitors

#### Everolimus

Everolimus can inhibit the mTORC1, and contributes to a high activation of the kinase AKT. Everolimus has important effect on the function of cell proliferation and the combination with azacitidine has shown promising clinical activity in AML [47].

#### Temsirolimus

Temsirolimus, a specific inhibitor of mTOR, interferes with cell growth. Currently, it is also found to be converted to sirolimus (rapamycin) in vivo [48]. Furthermore, the combination of temsirolimus with ibrutinib resulted in the cell growth reduction during the B-cell receptor pathway [49].

#### Sirolimus

Sirolimus, also known as rapamycin, inhibits activation of T cells and B cells. In vitro, it has also been found that sirolimus inhibits cell growth and even promotes cell death in B-precursor ALL [50, 51].

#### AZD8055

AZD8055 can inhibit mTORC1, mTORC2 and its downstream proteins through phosphorylation and markedly increase the survival of AML transplanted mice due to supress tumor growth [52]. Synergistic combinations of chemotherapy with low-dose AZD8055 could be more effective.

#### Ciclopirox

Ciclopirox, also an anti-fungal agent, is proved to be a novel specific mTOR kinase inhibitor and the combination with parthenolide which has been applied into preclinical anti-leukemia in AML and ciclopirox demonstrates greater availability against AML than treatment with either compound alone [53]. Besides ciclopirox could enhance the efficiency of compound parthenolide.

### MEK inhibitors

Pimasertib, a MEK1/2 inhibitor, is efficient to target many hematologic malignancies including ALL and AML, however, the probability of clinical benefit and more effective clinical trials still remains to be warranted [54, 55].

### AKT inhibitors

#### GSK690693

GSK690693 is a novel ATP-competitive Akt kinase inhibitor and selective for the Akt isoforms, while it also can inhibit additional members of the AGC kinase family [56].

#### Mk-2206

MK-2206 is another kind of oral inhibitor of Akt1/2/3 that promotes apoptosis and cell cycle arrest in AML [57]. During the trials of diffuse large B-cell lymphoma (DLBCL), MK-2206 significantly decreased p-AKT and downstream targets of AKT signaling [58].

#### T315I

T315I is an integrin-linked kinase (ILK) inhibitor, which downregulates protein kinase B (Akt) and p-Akt and decreases cell activity of AML [59]. The T315I is a unique mutation because of its resistance to the approved BCR-ABL inhibitors. The BCR-ABL fusion gene is a driver oncogene in chronic myeloid leukemia and 30–50% of cases of adult ALL [60, 61]. Introduction of ABL1 kinase inhibitors like imatinib has markedly improved patient survival, but drug resistance still remains a challenge.

### Other inhibitor

Alvocidib, also known as flavopiridol or HMR-1275, is a multi-serine threonine cyclin-dependent kinase inhibitor and mainly downregulates CDK7 and CDK9 to inhibit c-MYC, cyclin D1, and MCL-1. Alvocidib has currently been examined in a phase II study for the treatment of intermediate- and high-risk AML combined with othet effective agents [62].

## Potential inhibitors in acute leukemia

### Volasertib

Volasertib, also known as BI 6727, is a small molecule inhibitor of the polo-like kinase 1 (PLK1) protein, and being developed as an anti-cancer agent with the potential combination therapy for those untreated patients who are ineligible for intensive induction therapy [63]. Volasertib is currently undergoing investigation in phase I and II trials and has not yet been licensed by the FDA.

### Dasatinib

Dasatinib, former BMS 354825, is an orally available small-molecule multi-kinase inhibitor. It potently inhibits BCR-ABL and SRC-family kinases as well as PDGFR α/β, c-KIT, and ephrin receptor kinase [64]. Dasatinib is approved for the treatment of Ph + ALL resistant or intolerant to imatinib.

### BEZ235

BEZ235 is a dual PI3K/mTOR inhibitor and used in combination with dexamethasone in ALL. Inhibition of the PI3K/AKT/mTOR pathway with the dual PI3K/mTOR inhibitor BEZ235 enhanced dexamethasone-induced anti-leukemic activity both in vitro and in vivo models of T-ALL [65, 66].

### Idelalisib

Idelalisib is a promising treatment option for B-cell precursor acute lymphoblastic leukemia (BCP-ALL) patients with TCF3-PBX1 (E2A-PBX1), whereas other drugs could be useful depending on the genetic context of individual patients [67].

### PKI-587

PKI-587 is a selective inhibitor to suppress T-ALL cells proliferation and colony formation through PI3K/mTOR pathway. It has been reported that PKI-587 could delay tumor progression and enhance the survival rate during the mouse xenograft models [68].

### ABL001

ABL001, also named asciminib, could bind to the myristoyl pocket of ABL1 and induces the formation of kinase conformation. ABL001 is a potent and selective ABL1 inhibitor that is undergoing clinical development testing in patients with CML and Ph + acute lymphoblastic leukemia [69].

## Conclusions

In summary, molecular targeted therapy has demonstrated impressive efficacy and the development of PKIs has promising impact on acute leukemia patients. With the rapid development of biological information technology, multiple new types of PKIs have been selected to treat acute leukemia patients separately or jointly with traditional treatments. However, there are still some challenges to overcome, such as the off-target effects and stability of the PKIs, the mutations of protein kinases, the best dose for individual patient, the drug-resistance for PKIs, and the evoluted immune escape, etc. Thus, screening new targets and seeking novel effective PKIs are necessary and will provide more options for acute leukemia treatment.

## Abbreviations

ALL: Acute lymphoblastic leukemia
AML: Acute myelocytic leukemia
BCP-ALL: B-cell precursor acute lymphoblastic leukemia
BLK: B lymphocyte kinase
BTK: Bruton’s tyrosine kinase
C/EBP-α: CCAAT/enhancer-binding protein-α
CSF1R: Colony-stimulating factor 1 receptor
DLBCL: Iffuse large B-cell lymphoma
EGFR: Epidermal Growth Factor Receptor
FDA: Food and Drug Administration
FLT3: FMS-like tyrosine kinase 3
FLT3-ITD: FLT3 internal tandem duplications
FLT3-ITDs: FMS-like tyrosine kinase 3 internal tandem duplications
HDAC: Histone deacetylase
HSCT: Hematopoietic stem cell transplantation
ITD: Internal tandem duplication
JAK2: Janus kinase 2
JAK-STAT: Janus kinase signal transducer and activator of transcription
MAPK/ERK: Mitogenactivated protein kinase/extracellular signal regulated kinase
mTOR: Mammalian target of rapamycin
NPM1: Nucleophosmin
OS: Overall survival
PARP: Poly (ADP-ribose) polymerase
PDGFR α/β: Platelet-derived growth factor receptor α/β
PDGFRs: Platelet derived growth factor receptors
PDGF-Rs: Platelet-derived growth factor
Ph + ALL: Philadelphia-positive acute lymphoblastic leukemia
PI3K/AKT: Phosphatidyl-inositol 3-kinase/v-akt murine thymoma viral oncogene homolog 1
PKIs: Protein kinase inhibitors
PLK1: Polo-like kinase 1
RTK: Receptor tyrosine kinases
SCFR: Stem cell factor receptor
STAT5: Signal transducer and activator of transcription 5
TRK: Tropomycin receptor kinase
TrkA/B/C: Tropomyosin receptor kinase A/B/C
VEGFRs: Vascular endothelial growth factor receptors
